# Becoming our young people’s case managers: caregivers’ experiences, needs, and ideas for improving opioid use treatments for young people using opioids

**DOI:** 10.1186/s13011-022-00466-2

**Published:** 2022-05-07

**Authors:** Kirsten Marchand, Roxanne Turuba, Christina Katan, Chantal Brasset, Oonagh Fogarty, Corinne Tallon, Jill Fairbank, Steve Mathias, Skye Barbic

**Affiliations:** 1 Foundry, 915-1045 Howe Street, Vancouver, BC V6Z 2A9 Canada; 2 Faculty of Medicine, University of British Columbia, 317-2194 Health Sciences Mall, Vancouver, BC V6T 1Z3 Canada; 3 Department of Occupational Science and Occupational Therapy, University of British Columbia, 317-2194 Health Sciences Mall, Vancouver, BC V6T 1Z3 Canada; 4 Centre for Health Evaluation Outcome Sciences, 588-1081 Burrard Street, Vancouver, BC V6Z 1Y6 Canada; 5 Canadian Centre on Substance Use and Addiction, 75 Albert St #500, Ottawa, ON K1P 5E7 Canada; 6 Foundry Victoria, 818 Douglas St, Victoria, BC V8W 2B6 Canada; 7 Providence Health Care, St. Paul’s Hospital, 1081 Burrard St, Vancouver, BC V6Z 1Y6 Canada; 8 Providence Research, 1190 Hornby St, Vancouver, BC V6Z 2K5 Canada

**Keywords:** Adolescence, Young adult, Youth, Opioid use disorder, Opioid use, Caregivers, Human-centred co-design, Community-based participatory research

## Abstract

**Background:**

Evidence continues to show that young people, ages 15-24, remain at significant risk of harms from non-medical opioid use and opioid use disorder (OUD), with experts calling for widespread implementation of developmentally-appropriate interventions. These recommendations include the involvement of caregivers in the prevention, early intervention, and treatment of young people using opioids. However, little research has investigated caregivers’ experiences supporting young people, leaving critical gaps in understanding this role. The aim of this study is to explore caregivers’ experiences accessing opioid use treatments with young people and their needs and ideas for improving such treatments.

**Methods:**

This study reports qualitative findings from Phase 1 of the Improving Treatment Together project, a multi-phase, multi-site community-based participatory study broadly aimed at co-designing opioid use treatments to improve the experiences and outcomes of young people using non-medical opioids. During Phase 1, a total of 27 caregivers (parents, guardians) participated in full-day workshops that were conducted in three communities in British Columbia, Canada. Following human-centred co-design methods, caregivers engaged in small and large group discussions of their experiences, needs, and ideas for improving opioid use treatments for young people. Discussions were audio-recorded, transcribed verbatim, and thematically analysed.

**Results:**

Across communities, caregivers’ main experiences were defined as ‘becoming our young people’s case managers’ and ‘enduring a never-ending rollercoaster’. To improve these experiences, two needs themes were identified – expanding organizational and system-level capacity and wider-spread understanding of opioid use as a health issue. Caregivers brainstormed a total of 378 individual ideas to meet these needs, several of which spanned multiple needs themes.

**Conclusions:**

Caregivers’ experiences, needs, and ideas reveal critical opportunities for improving the quality of interventions for opioid use among young people. This study represents a substantial contribution to the design and implementation of developmentally-appropriate and family-centred interventions for young people using opioids.

## Background

It is well known that earlier initiation of any non-medical substance use is associated with progression to substance use disorders (SUDs) later in life and other related harms (e.g., co-occurring mental illnesses, injury, loss of social capital) [1–4]. These patterns are affected by a complex interaction between age-related factors (e.g., childhood adversity, family environment) and broader social and environmental contexts, such as substance use norms, and drug availability, which also evolve over time [5–8]. This is particularly worrisome considering the ongoing North American drug toxicity crisis, where highly contaminated opioid supplies have contributed to historically high mortality rates in young people (ages 15-24) [9–11]. In the United States (US), opioid-related mortality among young people has increased more than 3-fold from 1999 to 2018 [9, 10], with recent evidence suggesting an increased involvement of stimulants and other drugs in these rates over time [10]. In 2018, for instance, opioid-only mortality rates in young people were 0.19 per 100,000 individuals, while polysubstance-involved rates were 0.22 per 100,000 [10]. Although comparable national studies among young people are limited in the Canadian context, young people have accounted for approximately 20% of the total 24,626 opioid-related overdose deaths since 2016, when the crisis was declared a public health emergency [11]. Additionally, a recent population-based Canadian study reported an opioid-related crude mortality rate of 4.7 per 1000 person-years among young people receiving opioid agonist treatment (OAT) between 1996 to 2018 [12].

These data indicate that young people are a priority population in interventions for opioid use and opioid use disorder (OUD). However, research shows that young people receive inadequate assessment, diagnosis, and treatment for non-medical opioid use and OUD [13–16], leaving them at significant risk of harms, including fatal and non-fatal overdoses. To address this gap, experts have outlined evidence-based guidelines for developmentally-appropriate interventions [13, 17–20]. Briefly, these principles include early identification and intervention; a comprehensive approach to treatment (e.g., harm reduction, mental health, caregiver involvement, primary care) that aligns with young people’s individual goals; service environments that preserve young people’s autonomy and connection to community; continuous care and engagement, especially during times of elevated risk (e.g., relapse); and ongoing quality improvement efforts [13, 18].

Across guidelines, caregiver (e.g., parents, family members, guardians, family of choice) involvement is recommended to promote young people’s treatment engagement and outcomes [13, 18]. This recommendation is based on the protective effects that positive caregiver relationships have on young people’s substance use initiation and progression [21] and the effectiveness of family-based interventions (e.g., parent education, family counseling) in the prevention, early intervention, and treatment of young people’s substance use [21, 22]. Nevertheless, few studies have investigated the role of caregivers in opioid use treatment and services for young people [23]. In a recent review, Kaur et al. [23] identified two main foci in this scant literature, 1) ethical considerations and confidentiality when involving family members in young people’s opioid use treatment (*n* = 3 studies), and 2) the benefits of family involvement (*n* = 5 studies), including reduced rates of opioid use [24] and adherence to psychotherapeutic [25] and pharmacological treatments [26, 27].

Despite the valuable role and perspective that caregivers bring to improving developmentally-appropriate interventions for young people, very little research has explored their experiences in this role or ideas for such interventions. In one closely related study done prior to the current opioid-related drug toxicity crisis, Guarino et al. [28] conducted focus groups with parents to explore their perceptions of the effectiveness of methadone maintenance treatment for young people. Their findings showed that parents appreciated this treatment for its management of young people’s withdrawal symptoms and the opportunity to build new parenting strategies through the parent support groups. In a more recent study, caregivers discussed their limited understanding of the chronic and relapsing nature of OUD and their emotional exhaustion as factors influencing their ability to support their young people over time [29].

The present study adds to this limited body of research by further describing caregivers’ experiences accessing opioid treatment services with their young people across three communities in British Columbia (BC), a province that has faced some of the highest overdose death rates in Canada [11]. This work also adds original evidence regarding caregivers’ needs and ideas for improving the delivery of opioid use treatments for young people and families affected by non-medical opioid use. Our guiding research question was: *What are caregivers’ experiences, needs, and ideas for improving opioid use treatments for young people?* This research identifies opportunities and strategies for improving the quality of opioid use treatments and services for young people and responds to critical gaps in young people’s access to developmentally-appropriate and family-centred opioid treatment services.

## Methods

### Design, setting, participants

The Improving Treatment Together (ITT) Project is a multi-phase community-based participatory research (CBPR) project [30] that integrates human-centred co-design processes [31, 32], with the broader goal of producing actionable evidence leading to youth-centred opioid use treatments [33]. This paper focuses on qualitative data collected in Phase 1 of the project and follows consolidated criteria for qualitative research [34]. This phase involved a series of community-based workshops that were conducted between November 2019 and February 2020. At the time of project planning (2018), the project’s partners collaboratively identified three communities that were geographically diverse (i.e., spread across the province’s designated healthcare regions), varied in population size, and facing high regional rates of fatal opioid-related drug toxicity events, relative to the provincial rate (31.1 per 100,000) [35]. The three selected communities were Prince George, Victoria, and Vancouver, in BC, Canada. Briefly, Prince George is a smaller city (2016 population: 74,003) located in the Northern Health region and where fatal opioid-related mortality rates were 52.7 per 100,000. Victoria (2016 population: 85,795) is the provincial capital, located in the Island Health region of the province, and had 43.1 fatal opioid-related deaths per 100,000. Lastly, Vancouver is one of BC’s largest urban population centres (2016 population: 631,490), located in the Vancouver Coastal Health region, and where fatal opioid-related mortality rates were 57.1 per 100,000 [35].

To be eligible, participants were: (a) caregivers (i.e., biological parent, adopted parent, step-parent, guardian) of a young person between the ages of 16-24 who had current or past (within last 12 months) non-medical opioid use (e.g., heroin, fentanyl) and had accessed opioid use treatments; (b) able to speak and write in English; and (c) willing and able to provide fully informed consent to participate. To reach this sample, we relied on advertisements in collaborating community-based services for young people and families navigating substance use and snowball sampling. Interested participants then contacted the study team to verify the self-reported eligibility criteria.

### Procedures and data collection

Upon reviewing and signing the informed consent form, participants were asked to voluntarily complete the self-reported demographic questionnaire. Participants then engaged in a full-day workshop that was structured around the core elements of human-centred co-design [36]. These elements aimed to: (a) understand a caregiver’s experiences accessing opioid use treatments with their young person (empathy session); (b) identify and prioritize needs for improving opioid treatment service for young people (needs session); and (c) brainstorm solutions to address their selected needs (ideation session). These core elements were separated into structured workshop sessions.

During each session, participants self-selected into small discussion groups and were guided through broad open-ended questions, akin to focus group methods. In the first session, participants were prompted to share their experiences while accessing opioid use treatments with their young person. This session was guided by the open-ended questions, *“what are you thinking, feeling, hearing, saying, seeing, and doing during these point of care interactions?”.* After building a common understanding of each caregiver’s experiences, the next session focused on discussing and prioritizing needs, opportunities, and preferences for opioid use treatments for young people. In the ideation session, participants individually brainstormed solutions to the prioritized needs through broad open-ended questions (e.g., *“how might we … reduce waiting times?”*).

Each small group included 3-5 participants and two trained facilitators. Large group discussions were also used for wider reflection and clarification of similarities and differences between the small groups. The small and large group discussions for each session ranged from 30 to 90 minutes and all discussions were audio-recorded. Facilitators also took field notes and used white boards and worksheets to support data collection throughout each session. A community-based family peer-support team member was present at each workshop to provide on-site support and referrals, if needed. Participants were provided with catering, an honorarium for their time to participate, and reimbursement for travel-related expenses.

### Analysis

The primary source of data for the present analysis was the audio-recorded small and large group discussions, which were transcribed verbatim. Separate small (*n* = 25) and large group (*n* = 9) transcripts were created for each of the three discussion sessions (empathy, needs, ideas) and for each small discussion group. White board images (*n* = 25) were also used to support theme development. Field notes and worksheets were reviewed as part of the data quality check. The number of distinct data sources analysed from each community is shown in Table 1. All data sources were imported into NVivo [37] for analysis.

**Table 1:** Number of qualitative data sources used in the analysis from aross the three communities

Community	N participants per community	N small groups ^**a**^	N small group transcripts ^**b**^	N large group discussions & transcripts ^**c**^	N transcripts analysed	N whiteboard images ^**b**^
Prince George	6	2	6	3	9	6
Vancouver	8	3	9	3	12	9
Victoria	13	4	12	3	15	12
**Total number**	**27**	**9**	**25**	**9**	**36**	**25**

^a^Individual participants were separated into small discussion groups (akin to focus groups), ranging from 3 to 6 participants each

^b^Transcripts/white board images were separated by workshop session, i.e., one transcript/white board image for each of the empathy, needs, and ideas sessions

^c^One large group discussion was carried out for each of the three workshop sessions

Data from the experiences and needs session were thematically analysed following an inductive approach using Braun and Clarke’s six steps [38] by the first author (KM), who has extensive qualitative research experience. These steps began with repeated readings of the transcripts and supporting documentation. Next, initial coding was done by a data-driven approach (i.e., verbatim codes) and proceeded sequentially through each session of the workshop within each community. Initial codes from across all communities were then collated and sorted to search for potential semantic themes. Team meetings were held to review and discuss the relationships between codes and candidate themes (i.e., sub-themes, main overarching themes). Next, candidate themes and thematic diagrams from the empathy and needs sessions were discussed with four caregivers from the original workshops. These discussions informed theme definitions and names and strengthened the fit, interpretation, and connections of themes. In the final step, producing the report, the themes and selected extracts were presented back to the four caregivers for further feedback and refinement.

For the analysis of individual ideas collected during the ideation session, a blended theoretical and data-driven approach was selected. This approach was deemed most appropriate given the structured format of the workshops, where participants brainstormed as many ideas as possible to the identified needs. Additionally, the individual ideas were quite brief statements, which precluded a more in-depth analysis. After verbatim coding of individual ideas, the ideas were collated and sorted according to their fit with the final defined needs themes. As there were similarities between individual ideas coded under each needs theme, the ideas were then clustered into ideas themes using a data-driven approach.

## Results

### Participant characteristics

A total of 27 caregivers participated in the Phase 1 co-design workshops; 6 caregivers participated in the Prince George workshop, 8 in Vancouver, and 13 in Victoria. For further context, Table 2 displays participants’ socio-demographic characteristics and background data of their young people’s opioid use and treatment history. Participants primarily identified as woman (76%), Caucasian/White (75%), and having college/university degrees (57%). Participants first learned of their young person’s non-medical opioid use when the young person was an average of 17 years of age (standard deviation [SD] = 3.56). Forty-three percent of caregivers reported their young person’s daily use of opioids, 40% reported daily concurrent use of other non-medical substances (not including alcohol or cannabis), and 61% reported that their young person was currently using non-medical opioids. Individual counseling (e.g., cognitive behavioural therapy) was the most frequent opioid use treatment accessed by young people (86%), followed by case management (58%), and addictions medicine (57%). Thirty-eight percent of caregivers reported that their young person had received OAT (e.g., buprenorphine, methadone). Of note, 81% of caregivers reported that their young person had accessed more than one type of substance use treatment, with the mean number of different treatments being 3.7 (SD = 1.4).

**Table 2:** Characteristics of caregivers (*n* = 27) in three communities in British Columbia

Characteristic ^a^	N (%) / Mean ± SD
Number of participants in each community who responded to survey: ^a^	
Prince George	6 (22)
Vancouver	8 (30)
Victoria	13 (48)
Gender	
Woman	16 (76)
Man	5 (24)
Ethnicity	
Caucasian/White	15 (75)
First Nations, Inuit, Métis	4 (20)
Asian	1 (5)
Caregiver’s median age (Q1, Q3)	51 (47, 54)
Education	
Some college/university or less	9 (43)
College/university degree	12 (57)
**Characteristics of Young Person’s Substance Use**	
Age when caregiver first learned of their opioid use	17 ± 3.56
Frequency of non-medical opioid use during their period of use	
Daily	9 (43)
Weekly	7 (33)
Monthly	1 (5)
Unsure	4 (19)
Frequency of concurrent substance use during their period of opioid use (missing = 1) ^b^:	
None	2 (10)
Daily	8 (40)
Weekly	6 (30)
Monthly	1 (5)
Unsure	3 (15)
Currently using non-medical opioids (missing = 3)	
Yes	11 (61)
No	3 (17)
Unsure	4 (22)
Types of Substance Use Treatment Accessed ^c^:	
Counseling	18 (86)
Peer support	6 (29)
Case management	11 (58)
Psychiatry	7 (33)
Addictions medicine	12 (57)
Opioid agonist treatment	8 (38)
Private residential treatment setting	3 (14)
Accessed more than 1 type of these treatments	17 (81)
Mean number of different treatment types accessed	3.7 ± 1.4

*SD* standard deviation. Q1 = 25th percentile, Q3 = 75th percentile

^a^The socio-demographic survey was voluntary and was not mandatory to be able to participate in the workshops. Response rate to the survey was 78% (21/27 completed)

^b^In reference to non-medical substance use, not including alcohol and cannabis

^c^Participant could choose more than one type of treatment

**Fig. 1:**
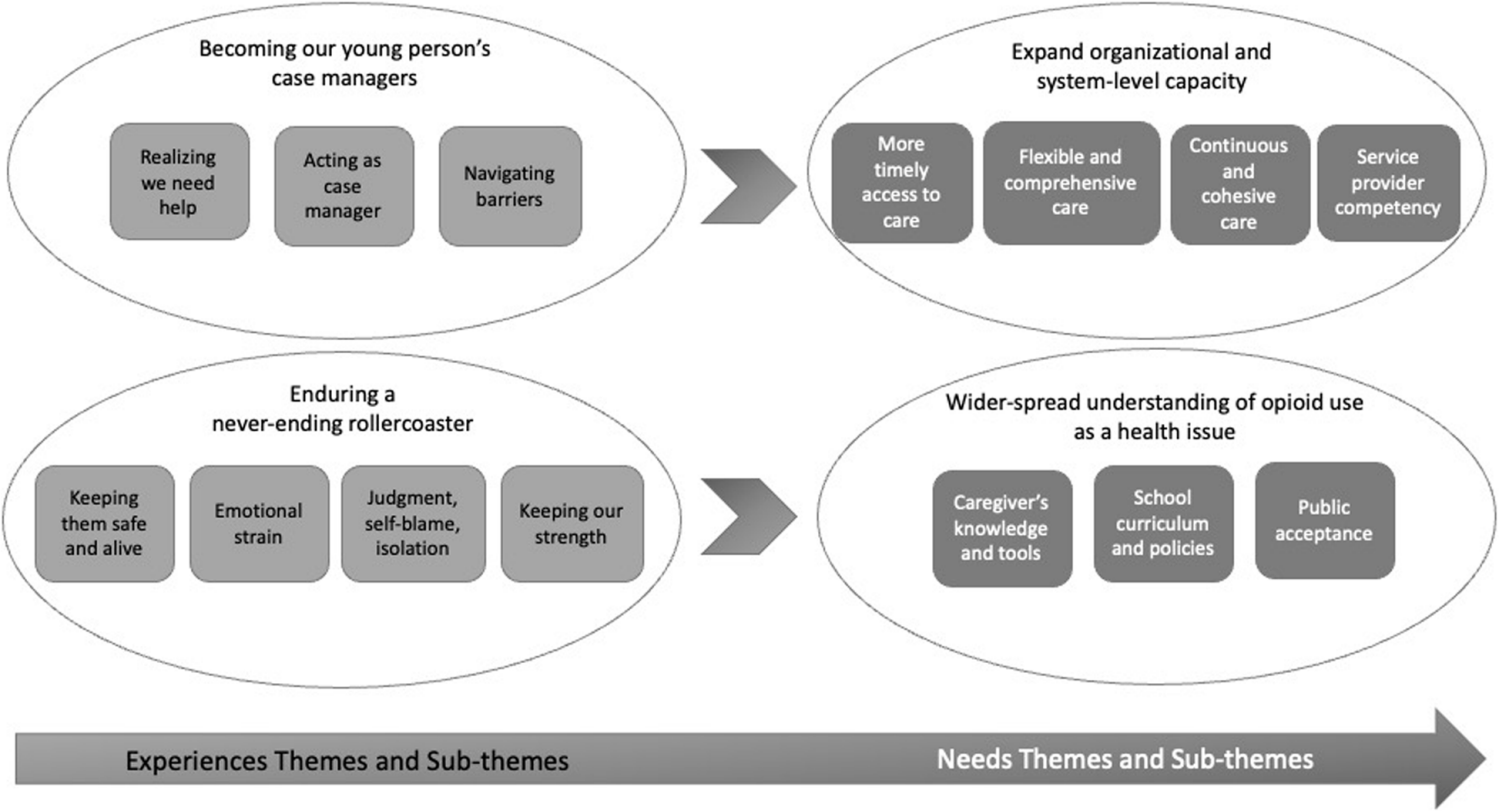
Caregivers’ experiences and needs for improving opioid use treatments for young people

The thematic analysis identified two main experiences themes and two main needs themes. These themes and their respective sub-themes are presented in Fig. 1. Given the flow of the workshop, there was a natural connection between the main experiences and needs themes, which is also illustrated in Fig. 1. The remaining sections summarize each of the main themes in greater detail, with single quotes identifying theme names and double quotes representing direct quotes from participants.

### Caregivers’ experiences accessing opioid use treatments with young people

#### Becoming our young person’s case managers

As participants supported their young person to access and keep connected to opioid use treatments, they shared the salient experience of ‘becoming our young person’s case managers’. This theme was contextualized by participants’ unique journeys as they first learned about their young person’s opioid use and realized that they needed help from service providers. For most participants, this was a gradual process as they initially thought their young person’s substance use was *“normal teenage behaviour”* (Caregiver in Victoria) and realized help was needed as they developed further knowledge of opioid use, OUD, and related harms. As one participant shared, most wished this realization had occurred much sooner:

*“I remember the moment where I realized it was time to ask for help, it was way down the road, it would've been so much better to have done so months or even years [earlier], but you know?... I know, at least for me, I didn't know enough to know what I was looking at, I couldn't see what was happening for a very long time. So that was part of the problem … I wish I would've started that years before I did.”* (Caregiver in Prince George)

As participants sought help, their experiences were depicted as *“scrambling, trying to find answers as fast as I can to somehow rescue them from this”* (Caregiver in Victoria)*.* In their efforts to support their young person, participants assumed many roles during this process. This included building their own knowledge of opioid use and related harms, leading the search for services, making daily phone calls to service providers and treatment and detox centres, *“connecting the dots for other providers”* (Caregiver in Victoria), and keeping connected to their young person while waiting for treatments. As one caregiver explained:

*“I think we’re given a job with the tools that are technically out there but without the power to access them [opioid use treatments] and it’s, it’s like spokes in a wheel, technically it’s all supposed to work together, but, but they don’t, they don’t necessarily all connect. So, we, we are really the only hub and yet we can’t access or um, [we have] all of the responsibility and none of the power.”* (Caregiver in Victoria)

Ultimately, participants explained that *“we become our own case managers”* (Caregiver in Victoria), which involved navigating many different spokes or systems that were not connected (primarily education, healthcare, and justice). Across these systems, participants recounted facing multiple barriers. These barriers clustered around accessibility issues, such as limited capacity for enrolment to services (e.g., not enough detox beds, OAT prescribers), narrow service delivery hours (i.e., weekdays from 9 to 5), and geographical variation in service options (e.g., services concentrated to a downtown core, limited OAT options in the north of the province). These issues increased the waiting time for services, disrupted treatment continuity between programs, and resulted in missed windows of opportunity where young people wanted treatment:

*“What I’ve noticed a lot from my experience, um, when you’re using [opioids], and you don’t want help that’s when they’ll [service providers] tell the parents, like ‘oh you just have to wait until they want help. You can’t really do anything until they are willing to accept it’. But then when he [son] wanted help, that’s when it’s ‘oh you’re not stable enough, oh there’s a wait list, oh you can’t come here, you can’t do this, you can’t do this’. And it’s like they keep telling the parents, at least from my perspective um, you need to wait until they are actually willing to accept help, but then when they’re willing to accept help, they don’t get it, or they don’t get it fast enough. And then, they start to feel like there’s no point and then they start using again … it’s like you’re waiting for them to want help and then they want help, they don’t get it.”* (Caregiver in Victoria)

As this quote showed, participants attributed some of these accessibility issues to systems that were *“reactive rather than proactive”* (Caregiver in Prince George)*.* This left caregivers and young people with few timely options during those critical windows, and few supports to address the *“reasons why they started using [opioids] in the first place”* (Caregiver in Prince George), such as trauma, pain, and co-occurring mental health symptoms.

Barriers to treatment access and continuity were also rooted in age-based treatment policies (e.g., British Columbia’s Infant Act), as many organizations use young people’s biological age (commonly 19 or 24) to define the transition between child/youth and adult healthcare systems [39]. These age-based transitions meant that young people had to “*start all over just because they turned 19”* (Caregiver in Vancouver)*,* disrupting service continuity and trusted service provider relationships. These policies also affected the extent to which caregivers could be involved in young people’s treatment engagement, when appropriate and preferred:

*“[For] someone who’s over the age of 19, you know, having services that involve the family would make a big difference, right? There’s this expectation now that he’s the age that he can navigate this all on his own, right? Um, you know, for like – like psychiatry appointments and his programs and making sure his forms are filled out … but a lot of it I still manage … so having that ability to connect right with his service providers as his circle of care, instead of expecting him to be able to manage that, would make a huge difference. Yeah, it’s like 18 all the support and 19, you’re on your own right? And it just, it doesn’t make sense? At 45 I think I can manage most of it, but I can’t imagine in your early 20s, you know, [with] concurring instances of mental health and addiction, and treatment, you know?”* (Caregiver in Prince George)

#### Enduring a never-ending rollercoaster

The second experiences theme (Fig. 1) was rooted in participants’ drive to keep their young people safe and alive. As this caregiver describes, this led to “*horrific*” situations:

*"You slowly ease yourself into it because it's a nightmare, it's so horrific that it blows my mind, you know? There's times we walked into the room, and he's blue, he's not breathing, his heart's not going, you know we're doing CPR [cardiopulmonary resuscitation] and I’m injecting him [with naloxone] and I’m just trying to hold it together while I’m trying to revive my son. And it's like, I can't live like this, and yet, I have to. So, we actually kicked him out years ago … um, but then as it got towards the fall and winter and stuff, his usage got so much, I had to ask myself the question, you know, am I willing to receive that phone call that says ‘sorry sir your son's dead’? And I couldn't, I was, I had to take him home, the whole firm hand thing? I just threw that out the window. I had to keep him alive."* (Caregiver in Victoria)

As this participant’s experience depicts, this never-ending rollercoaster was emotionally straining as it evoked constant fear, helplessness, hopelessness, and frustration. Participants also experienced self-blame, shame, and isolation due to judgment, stigma, and lack of understanding of opioid use. As this caregiver explained:

*“And then we have people that you think you can talk to, and they say, ‘oh well, that’s her choice’. It’s so frustrating because it’s like … but don’t you understand – even people who have had it in their family, they say that, and it’s just like… it takes over your whole life, it’s like air. They don’t get it, they don’t understand … And it’s just very frustrating … you want the whole family to be there supporting her and trying to, you know, do everything we can. But every time you think you take a step forward, other people are just tearing her down.”* (Caregiver in Vancouver)

To maintain their strength through their roles as case managers, participants emphasized the importance of connecting with other families with lived/living opioid use experience:

*“I am not as ashamed, or embarrassed, or as stigmatized anymore … I have found some good support groups that I've gone to. One was a biweekly parent meeting. I've gone to SMART Family and Friends … Personal counselling, that's been helpful. So, I think it's just been a process, but it's been a process with a lot of peer support.”* (Caregiver in Vancouver)

### Caregivers’ needs for improving opioid use treatments for young people

Figure 1 also shows the two main needs themes, which are further described in the following sections.

#### Expand organizational and system-level capacity

Building on their main experience of ‘becoming our young person’s case managers’, participants emphasized the need for expanded organizational capacity among specialized service providers and treatment programs so that young people can get timely access to services during windows of opportunity. Participants also described that organizations needed to adapt their policies and procedures to reflect young people’s diverse histories (e.g., root causes, concurrent mental illnesses), current circumstances, and long-term goals (e.g., arts, mentorship, vocational programs):

***Participant 3:*** *Um, I think with youth, you kind of get more opportunity to really make an impression, especially if you’re a brand new service that they’ve never accessed before, and if they don't connect with you that's kind of probably the end of it. Um, and if they're not met very warmly, like if, if you're told ‘oh come back in an hour’, or um, ‘we don't do that anymore’ or ‘you're in the wrong place’. They’re not going to go back …*

***Participant 1:*** *And just in a timely way... Just like expanding hours.*

***Participant 2:*** *Yeah, it needs to be responsive in a way that meets the need at the time, not a couple of days from now.*

***Participant 3:*** *Four days isn’t fast.*

***Participant 4:*** *… And, I think an opportunity could be that adaptation of expectations when young people are accessing treatment or services, right? Like we talked about this a little bit on the break, you know that expectation of abstinence, right? Is that realistic? Probably not. We know, and research shows that isn't the expectation, right? So, then you take away, you know, the substance they were using to get by, plus also the coffee and the cigarettes and then stick them in a really unfamiliar place with unfamiliar structures, that's really unrealistic right?* (Caregivers in Prince George)

Participants also discussed the need for a more cohesive system, with emphasis on strengthening the consistency of policies and operating procedures (e.g., intakes, caregiver involvement, expectations of abstinence) among different systems, organizations, and professionals. Participants also identified the need for increased competency in substance use treatment among service providers throughout the healthcare system, and not just in specialized settings. Similarly, caregivers reflected on the need for better communication and information sharing between different service providers and systems and having “*somebody that’s familiar with the police … the medical system and what they have to offer … I mean there’s so many different systems and you have to kind of learn all of them”* (Caregiver in Victoria)*.* As this quote suggests, these needs were closely connected to the difficulties participants experienced navigating different systems (e.g., healthcare and police; child/youth and adult systems).

#### Wider-spread understanding of opioid use as a health issue

The second theme reflected an underlying need for *“less stigma in our community”* (Caregiver in Victoria) and increased recognition that opioid use (and substance use and mental health generally) is a health or medical issue, rather than a criminal one. As shown in this exchange between caregivers in Prince George, participants discussed that this recognition could lead to increased opportunities for intervention and less stigma:

***Participant 2:*** *We would treat a physical illness much differently than we would treat a mental [health] challenge, but they can overlap, and they can fall in the same model. And yeah, we don't look at it that way …*

***Participant 3:*** *I have redefined it in my mind, I feel like my son has something that's sort of like cancer and if he doesn't get the right treatment, he might die. And that's how I see it. And I know he's doing the best that he can. But, the rest of us could be doing a little bit better, because like you say, if he did have cancer, man, we'd be [using] a different approach and no one would be saying you have to stop doing this. You know? …*

***Participant 1:*** *This is a medical disorder … They need proper care.*

***Participant 3:*** *… I think the medical discourse is more acceptable so people can get behind that and stop judging people because it’s medical. I’m not sure, I don’t know, right? It is psychological too, it comes from pain, it comes from trauma, there’s other things involved … I think talking about it medically is useful because it gets us away from the blaming the person and the stigma … I think we have an opportunity here to change the culture.*

This need reflected a deeper understanding of substance use as a health issue on both individual and societal levels and, thus, was discussed in relation to multiple groups, including caregivers and family members, schools and educators, and the wider public. Among individual caregivers, this theme was connected to the sub-theme ‘realizing we need help’ and needing more knowledge, caregiving skills, and whole family support to better care for their young person. In school settings, participants discussed the need for earlier introduction of evidence-based curriculum on mental health and substance use. Participants also stressed that this curriculum be taught *“from a science point of view”* (Caregiver in Vancouver) and that school policies be less punitive (e.g., remove expulsion for using substances) so that young people can maintain their connections to schools, improve their understanding of the risks and harms associated with opioid use, and experience less stigma.

### Ideas for improving opioid treatment services for young people

Across the three workshops, a total of 378 individual ideas for improving opioid use treatments were identified by caregivers. Table 3 presents the ideas themes and select representative individual ideas that were identified within each of the needs themes. For the first main needs theme, ‘expand organizational and systems-level capacity’, the sub-theme ‘continuous and cohesive care’ had the highest number of individual ideas. Within this sub-theme, the most frequent ideas centred around solutions to maintain consistency in service providers for young people across their service journey. For instance, individually assigned case managers or advocates who would be with young people over time, supporting treatment access, follow-up, and transitions. For the needs sub-theme, ‘flexible and comprehensive care’, peer connections across the journey (e.g., peer mentoring programs) were frequently identified as solutions to better meet young people’s diverse needs over time. For the needs sub-theme, ‘more timely access to care’, the most frequent ideas centred around increasing the number of available beds or spaces for young people in detoxification, treatment, and stabilization programs/centres. For the needs sub-theme, ‘service provider competency’, participants identified training in active listening, non-judgment, and cultural safety as key solutions.

**Table 3:** Caregivers’ ideas for improving opioid use treatments for young people by needs themes

Caregiver Needs Themes ^**a**^	N ^**b**^	Ideas Themes ^**c**^	Representative Individual Idea ^**d**^
**Main Need Theme: Expand organizational and systems-level capacity**			
1. More timely access to care	62	• Increase availability of beds for detox, treatment, and stabilization (*n* = 21)	“More beds available in stabilization and treatment”
		• Centralized information about service options, requirements, and current wait times (*n* = 12)	“Create a service map showing all the resources and supports available to youth using opioids”
		• Instant access to peer-based services (*n* = 7)	“Instant access to peers at hospitals”
		• Flexible service delivery hours (*n* = 7)	“Change service hours. 9:00 am is quiet so instead of 9-4, change to 11 am-7 pm”
		• Instant access to navigators (*n* = 5)	“A person that can help parents navigate all the systems, point them in the right direction, give contacts”
		• Assign addictions teams at critical events (e.g., first hospitalization, overdose, arrest, etc.) (*n* = 4)	“Every kid should have a care team. If they overodose, or you have police come to your house, you get this team of people assigned”
		• E-health interventions (*n* = 3)	“An app for kids to communicate with professionals discreetly”
		• Drop-in and on-call services (*n* = 3)	“On-call youth and family advocates”
2. Flexible and comprehensive care	73	• Peer connections across the journey (*n* = 17)	“Peer mentoring programs for youth with substance use disorders”
		• Family involvement in treatment plans (*n* = 13)	“Service providers to revisit with youth on a continuous basis how they would like family involved”
		• Provide services that reflect youths’ developmental needs (*n* = 10)	“Expand concept of treatment plan to nature, outings, art, etc. where appropriate”
		• Create inclusive and comfortable environments (*n* = 10)	“Comfortable environments, ambient lighting, calming images, snacks, fidgets”
		• Integrated services for mental health, substance use, and life skills training in substance use treatment (*n* = 9)	“Build in vocational training programs that are flexible”
		• Co-create treatment plans with youth and update them frequently (*n* = 7)	“Goals based on youth and frequently updated as stage changes”
		• Increase harm reduction programs for youth (*n* = 7)	“Drug checking services”
3. Continuous and cohesive care	75	• Maintain consistent service providers for youth throughout their journey (*n* = 43)	“Attach a consistent case manager or advocate to link services and follow-up”
		• Create smoother transitions between treatment types (*n* = 15)	“Treatment centres next door to detox centres”
		• Provide services based on developmental needs, not age (*n* = 10)	“Criteria for services based on need, not age (e.g., hierarchy of needs)”
		• Set up ‘one-stop shop’ for youth (*n* = 5)	“Multiple appointments ongoing in same space to work towards collective plan of action”
		• Clearer lines of collaboration and communication between supports (*n* = 2)	“Clear lines of communication between all parties of support, including service providers (emergency, social services, etc.), youth, and families”
4. Service provider competency	12	• Training in active listening, non-judgment, cultural safety (*n* = 9)	“Service providers who hold space with someone”
		• Training in how to involve families (*n* = 1)	“Training for service providers in how to involve families in treatment plans”
		• First responder education in youth and opioid use (*n* = 1)	“Develop education around youth and opioid problems and unsafe for first responders”
		• Support for service providers (n = 1)	“Better pay and support for service providers”
**Main Need Theme: Wider-spread understanding of opioid use as a health issue**			
1. Caregivers’ knowledge and tools	107	• Programs that connect parents with others with similar experiences (*n* = 45)	“Create a network of parents of youth using opioids to share information and resources with each other”
		• A handbook or guide of community resources (*n* = 26)	“Parent manual or courses on how to raise a teen with a substance use disorder”
		• Resources or courses that prepare parents and families for what to expect and next steps (*n* = 14)	“On first overdose, resources that tell me what the next steps are and what to expect”
		• Websites for parents with resources and information (*n* = 11)	“Website that has all of the services, their requirements and is updated often”
		• Wider- spread information and resources (*n* = 8)	“Distribute info through family doctors and youth clinics”
		• Teams that help caregivers with service navigation (*n* = 3)	“Parent navigator or advocate at each substance use service”
2. School curriculum and policies	44	• Integrate mental health and substance use liaisons in each school (*n* = 16)	“Mental health professionals on-site or on-call at schools”
		• Peer-based mental health and substance use programs in schools for students and staff (*n* = 11)	“Develop peer-based substance use education programs in academic and school environments”
		• Increase mental health and substance use training for school professionals (*n* = 10)	“Empathy training for all adults in education system that is specific to mental health and substance use”
		• Integrate curriculum on mental health and substance use (*n* = 7)	“Bi-annual curriculum specific to mental health and substance use”
43. Public acceptance	5	• Tackle stigma in the community (*n* = 3)	“Make it okay for people to say ‘I’m not okay’”
		• Public awareness campaigns (*n* = 2)	“Make a big campaign spreading information and spreading empathy”

^a^Needs correspond to the sub-themes presented in Fig. 1, which were used to guide sorting of individual ideas from across all three workshops

^b^Number of individual ideas coded at each sub-theme

^c^Ideas were brainstormed by individual participants in the workshops and were documented on flip charts. Data shown reflect the semantic ideas themes, which represent clusters of individual ideas that were similar across participants and workshops. Data in the brackets represent the number of individual ideas that were collated into the idea theme, thus a higher number represents a higher number of individual ideas coded in the respective ideas theme

^d^Data shown are representative examples of individual ideas that were coded within the semantic ideas themes

For the second main needs theme, ‘wider-spread understanding of opioid use as a health issue’, the sub-theme ‘caregivers’ knowledge and tools’ had the highest number of individual ideas coded. Among these, two key ideas included programs that can connect parents with other caregivers who have similar experiences and handbooks or guides. With both of these ideas, there was emphasis on these ideas allowing for knowledge and resources to be shared, while simultaneously building a community of support. For the ‘school curriculum and policies’ needs sub-theme, the most frequently identified ideas included the development and implementation of peer-based substance use programs in schools for students and staff as well as increasing mental health and substance use training for school professionals. Lastly, there were few specific ideas identified for the needs sub-theme ‘public acceptance’, with public awareness campaigns being most actionable.

Of note, several similar ideas themes were identified from across the needs sub-themes, suggesting their potential importance to addressing multiple caregiver needs. Peer-based services for youth (e.g., instant access to peer-based services) and caregivers (e.g., programs that connect parents with others with similar experiences) were solutions that were identified for four of the seven needs sub-themes. Similarly, assigned addictions care teams, system navigators, and liaisons were also conceptually similar ideas that spanned multiple needs. Finally, integrated mental health and substance use services and one-stop shops were also overlapping ideas across the needs themes for ‘flexible and comprehensive care’ and ‘continuous and cohesive care’.

## Discussion

This study reports caregivers’ experiences, needs, and ideas for improving opioid use treatments for young people based on data collected during Phase 1 of the multi-phase and multi-site Improving Treatment Together (ITT) project. This research responds to critical health services-, advocacy-, and policy-related gaps by identifying targeted youth- and family-centred solutions that evolved directly from caregivers’ experiences and needs.

### Caregivers experiences

Across the three communities, participants’ main experiences were defined as ‘becoming our young person’s case manager’, which involved navigating multiple roles and systems while ‘enduring a never-ending rollercoaster’. These experiences can be further contextualized in the findings from the socio-demographic survey. At the time of the workshops, 61% of caregivers reported that their young person was currently using non-medical opioids, and during periods of regular opioid use most young people were using weekly or daily and concurrently using other substances (other than alcohol and cannabis). Caregivers also reported that their young person accessed an average of 3.7 different types of treatments, primarily counseling and case management, with 38% reporting OAT. These findings follow recent large-scale studies of young people’s patterns of opioid and polysubstance use [10, 40, 41] and treatment utilization, where young people primarily receive psychotherapeutic interventions [13, 15]. These data also support understanding of the experiences themes, as caregivers’ described navigating multiple systems while trying to keep their young people safe amidst the current drug policy crisis of highly contaminated opioid supplies [9–11].

The in-depth exploration of caregivers’ experiences also revealed that they endured significant burdens in the caregiver role. In particular, they described having *“all of the responsibility”* (Caregiver in Victoria) but limited decision-making power and knowledge to effectively navigate multiple siloed systems. These findings complement a recent study where caregivers emphasized tensions between maintaining their supportive caregiving roles while respecting young people’s autonomy [29]. Our findings also support a growing body of research that is concerned with understanding the burdens associated with SUD-related caregiving roles [42–44]. For example, a recent study of OUD-caregivers (primarily a parent, child, or spouse) found that caregivers’ income, stress, and stigma were predictors of burden, while their personal mental and physical health were predictors of resilience [44]. These findings are highly relevant to recent recommendations for developmentally-appropriate principles of care for young people who use substances [13]. Of relevance to our study is the common recommendation to include family members as potential sources of support in the assessment and intervention of harmful opioid use among young people [13, 18, 45]. Based on this growing body of research, it is critical that further attention be directed towards how to uphold these recommendations while not overburdening caregivers.

### Caregiver needs and solutions

Through our innovative use of human-centred co-design, caregivers in the present study identified several solutions to better meet their prioritized needs. Recurring ideas across communities focused on the development of peer support networks and manuals on how caregivers can best support young people using opioids. As shown by our study and others [44, 46, 47], such solutions could promote caregivers’ knowledge and tools, resilience, and advocacy for more timely and appropriate interventions and policies. Participants also emphasized the need to expand organizational and systems-level capacity so that they could focus on being a parent/guardian instead of *“connecting the dots”* (Caregiver in Victoria) between service providers. Here, caregivers expressed the need for care to be timely, comprehensive, and continuous. Of note, these needs also strongly overlap with several of the recent developmentally-appropriate principles of care for young people [13, 18]. For instance, these guidelines recommend comprehensive assessment and treatment of young people’s substance use, concurrent mental illnesses, physical health, and psychosocial needs [13, 18]. However, our findings indicate that there remain significant gaps in the delivery of such comprehensive and continuous care, as caregivers described a lack of services addressing the *“root causes”* (Caregiver in Prince George) of young people’s opioid use, concurrent mental illnesses, and their long-term goals.

To address these gaps in services, caregivers identified system-level solutions, such as integrated mental health and substance use services and one-stop shops. These ideas align with innovative integrated youth service (IYS) hubs (e.g., Foundry in BC, Youth Wellness Hubs Ontario, allcove in California, Jigsaw in Ireland, headspace in Australia), which are increasingly being implemented to address longstanding challenges with the appropriateness and accessibility of youth mental health and substance use service provision and engagement [48]. These hubs aim to provide young people (typically ages 12-24) with rapid access to comprehensive support, including mental health, substance use, primary care, social, and peer support services in integrated, community-based, and youth-friendly settings [48, 49]. Emerging evidence suggests that IYS hubs are highly endorsed by youth [50], caregivers [51] and service providers [52], with further research on their effectiveness in the assessment and treatment of harmful substance use highly anticipated [49].

Innovative IYS hubs may also be uniquely positioned to develop the strategies and resources that caregivers identified for their prioritized need of ‘wider-spread understanding of opioid use as a health issue’. This needs theme targeted distinct groups that frequently interact with young people, including caregivers, schools, and community-based service providers. To meet this need, caregivers’ identified several solutions, such as education for schools and first responders on identification of substance use and best practices. As IYS models are based on principles of youth and family engagement and interprofessional collaboration [48], they are well-positioned to promote developmentally-appropriate strategies and interventions through partnerships with schools, service providers, and policymakers.

### Future directions

Based on this set of findings, Phase 2 of the ITT project is currently co-developing health service innovations to meet caregivers’ prioritized needs [33]. This has involved an extensive selection and co-design process. After the Phase 1 workshops, the full set of solutions underwent review by the project’s community, provincial, and national partners. The solutions were appraised against a set of a selection criteria for their impact, feasibility, scalability, and sustainability [33]. Since then, a working group, comprised of caregivers and the project team, has been working collaboratively to co-design selected solutions. Results of the co-design process, final prototypes, implementation, and evaluation are forthcoming.

### Limitations

To our knowledge, this is one of the first studies to explore caregivers’ experiences, needs, and solutions for improving young people’s opioid use treatment access and outcomes. However, these findings must be interpreted with consideration of the limited diversity of participants’ characteristics when recruited through community-based organizations offering family support services for youth substance use. Across workshops, we reached a sample of caregivers that primarily self-identified as Caucasian/White and with higher education levels. Thus, our findings may not be applicable to caregivers and young people facing further structural inequities (e.g., racialized communities, low socio-economic status) that affect opioid use and treatment patterns and experiences [53, 54]. Future studies may consider using sampling strategies, such as maximum variation, that will promote greater representation of these underrepresented groups.

There were also important lessons from our application of human-centred co-design to this research study. While this method was valuable at identifying solutions that were grounded in caregivers’ experiences, it is a highly iterative method that brings a unique set of considerations. Of relevance to our data, participants were able to move between different small discussion groups within their community’s workshop as their interests evolved. This made it difficult to maintain unique identifiers across small group discussion sessions and precludes our integration of the thematic findings with participant’s individual characteristics (e.g., caregiver ethnicity, number of times young person accessed treatment) that would further support interpretation. Future researchers considering this approach should apply procedures that enable consistency in participants’ identifiers across all data sources, while still ensuring anonymity.

## Conclusions

Our multi-site qualitative findings revealed that caregivers undertake a significant role in young people’s opioid use treatment engagement. This supports recommendations to involve caregivers in the delivery of developmentally-appropriate interventions (where appropriate and preferred). However, caregivers’ experiences and needs revealed critical opportunities for improving these interventions and caregivers’ roles. Caregivers identified several timely, feasible, and impactful solutions to improve these experiences and reduce opioid-related harms in young people. These solutions are critical to service providers and policymakers seeking further innovations in the design and delivery of developmentally-appropriate and family-centred approaches to opioid use and OUD.

## Data Availability

The datasets generated and/or analysed during the current study are not publicly available due to potential for identifying participants but are available from the corresponding author on reasonable request.

## Abbreviations

OUD: Opioid use disorder
SUD: Substance use disorder
ITT: Improving Treatment Together project
CBPR: Community-based participatory research
BC: British Columbia
SD: Standard deviation
OAT: Opioid agonist treatment
